# Arrhythmogenic Right Ventricular Cardiomyopathy Post-Mortem Assessment: A Systematic Review

**DOI:** 10.3390/ijms25052467

**Published:** 2024-02-20

**Authors:** Vincenzo Cianci, Elena Forzese, Daniela Sapienza, Alessio Cianci, Antonio Ieni, Antonino Germanà, Maria Cristina Guerrera, Fausto Omero, Desirèe Speranza, Annalisa Cracò, Alessio Asmundo, Patrizia Gualniera, Cristina Mondello

**Affiliations:** 1 Department of Biomedical and Dental Sciences and Morphofunctional Imaging, Section of Legal Medicine, University of Messina, Via Consolare Valeria, 1, 98125 Messina, Italy; elena.forzese.94@gmail.com (E.F.); daniela.sapienza@unime.it (D.S.); alessio.asmundo@unime.it (A.A.); patrizia.gualniera@unime.it (P.G.); mondelloc@unime.it (C.M.); 2 Department of Cardiovascular Medicine, Fondazione Policlinico Universitario A. Gemelli-IRCCS, Largo A. Gemelli 8, 00168 Rome, Italy; 3 Department of Human Pathology in Adult and Developmental Age “Gaetano Barresi”, Section of Pathology, University of Messina, 98125 Messina, Italy; antonio.ieni@unime.it; 4 Zebrafish Neuromorphology Lab, Department of Veterinary Sciences, Via Palatucci Snc, University of Messina, 98168 Messina, Italy; agermana@unime.it (A.G.); mariacristina.guerrera@unime.it (M.C.G.); 5 Medical Oncology Unit, Department of Human Pathology “G.Barresi”, University of Messina, 98125 Messina, Italy; faustoomero@hotmail.it (F.O.); desiree.speranza@gmail.com (D.S.); 6 Department of Biomedical Sciences and Morphological and Functional Imaging, Diagnostic and Interventional Radiology Unit, University Hospital Messina, 98125 Messina, Italy; annalisacraco@hotmail.it

**Keywords:** arrhythmogenic right ventricular cardiomyopathy, post-mortem analysis, post-mortem genetic test, cardiomyopathy, family screening for cardiomyopathies, sudden cardiac death

## Abstract

Arrhythmogenic right ventricular cardiomyopathy (ARVC) is a genetic disorder characterized by the progressive fibro-fatty replacement of the right ventricular myocardium, leading to myocardial atrophy. Although the structural changes usually affect the right ventricle, the pathology may also manifest with either isolated left ventricular myocardium or biventricular involvement. As ARVC shows an autosomal dominant pattern of inheritance with variable penetrance, the clinical presentation of the disease is highly heterogeneous, with different degrees of severity and patterns of myocardial involvement even in patients of the same familiar group with the same gene mutation: the pathology spectrum ranges from the absence of symptoms to sudden cardiac death (SCD) sustained by ventricular arrhythmias, which may, in some cases, be the first manifestation of an otherwise silent pathology. An evidence-based systematic review of the literature was conducted to evaluate the state of the art of the diagnostic techniques for the correct post-mortem identification of ARVC. The research was performed using the electronic databases PubMed and Scopus. A methodological approach to reach a correct post-mortem diagnosis of ARVC was described, analyzing the main post-mortem peculiar macroscopic, microscopic and radiological alterations. In addition, the importance of performing post-mortem genetic tests has been underlined, which may lead to the correct identification and characterization of the disease, especially in those ARVC forms where anatomopathological investigation does not show evident morphostructural damage. Furthermore, the usefulness of genetic testing is not exclusively limited to the correct diagnosis of the pathology, but is essential for promoting targeted screening programs to the deceased’s family members. Nowadays, the post-mortem diagnosis of ARVC performed by forensic pathologist remains very challenging: therefore, the identification of a clear methodological approach may lead to both a reduction in under-diagnoses and to the improvement of knowledge on the disease.

## 1. Introduction

Sudden cardiac death (SCD) is defined as a “sudden and unexpected death occurring within an hour of the onset of symptoms, or occurring in patients found dead within 24 h of being asymptomatic and presumably due to a cardiac arrhythmia or hemodynamic catastrophe [1]”. In the United States, SCD represents a leading cause of death and it is estimated to be responsible for up to 450,000 deaths every year [1]. Approximately 70% of SCDs are related to coronary artery disease, 15% to cardiomyopathies, 5% to valvular disease, 2% to inherited arrhythmia syndromes and 8% to other causes [1,2]. 

However, considering only SCDs in patients up to the age of 35, there is a substantial reduction in cases related to coronary heart diseases by up to 24%, but, nevertheless, the percentage of SCD caused by cardiomyopathies remains stable at around 16%. Indeed, interestingly, about 40% of the SCD etiology within this age group remains unknown: in these cases, a more extensive investigation and comprehensive post-mortem genetic tests could reduce the number of missed diagnoses and may guarantee the correct identification of genetic disorders with a negative histological phenotype, including some forms of arrhythmogenic right ventricle cardiomyopathy (ARVC) [2]. Indeed, ARVC is a genetic disorder that may present with a broad clinical spectrum, ranging from sporadic palpitation to an abrupt onset with sustained ventricular arrhythmias leading to sudden cardiac death (SCD), which may be the first manifestation of an otherwise silent pathological condition. Nevertheless, currently, there are no universally accepted autoptic criteria to formulate a certain diagnosis of ARVC becoming its correct identification is even more complicated in cases where the ventricular myocardium does not show typical macroscopic or microscopic signs of the pathology [3,4]. 

From a nosographic perspective, ARVC belongs to the family of cardiomyopathies. Cardiomyopathies are a large and heterogeneous group of pathologies affecting the heart muscle, which are “*structurally and functionally abnormal, in the absence of any other cause sufficient to determine the observed myocardial abnormality*” [3]. According to the recently published European Society of Cardiology (ESC) guidelines, in 2023, cardiomyopathies were classified considering the morphological and functional characteristics of the myocardium into five categories: hypertrophic cardiomyopathy (HCM), dilated cardiomyopathy (DCM), non-dilated left ventricular cardiomyopathy (NDLVC), arrhythmogenic right ventricular cardiomyopathy (ARVC) and restrictive cardiomyopathy (RCM) [3,5]. 

Before the development of both genetic tests and cardiac magnetic resonance (CMR), arrhythmogenic cardiomyopathy of the right ventricle/dysplasia had been defined as a pathology predominantly affecting the right ventricle, typically manifesting with malignant ventricular arrhythmias [6]. Then, the advancement of autopsy techniques, the increase in genetic knowledge underlying the pathology and the growing use of CMR with contrast enhancement have allowed for a better identification of the fibro-adipose alterations of the cardiac muscle, which is not limited exclusively to the right ventricle, but could affect both cardiac chambers through different mechanisms [6].

Therefore, the scientific community had coined a new name to identify this pathology, exclusively defined as “arrhythmogenic cardiomyopathy” (ACM) [7]. Nevertheless, since arrhythmias remain the main complication of this pathology and they are not attributable to a specific phenotype, the use of this new definition as a distinct subtype of cardiomyopathy was not recommended, as it is not consistent with the existing classification scheme. Therefore, while “ACM” is recognized as a generic term that encompasses several clinical phenotypes, it has been sought to improve this classification to differentiate all clinical phenotypes and to evaluate the different genotype–phenotype correlations [8,9].

To overcome all these problems, as mentioned above, the European Society of Cardiology (ESC) has proposed a new phenotype-based classification for cardiomyopathies, responding to the evidence of the genetic and clinical overlap between right and left ventricular arrhythmogenic cardiomyopathies [3]. ARVC can therefore be defined as “*the presence of predominantly right ventricle dilatation and/or dysfunction in the presence of histological involvement and/or electrocardiographic abnormalities, in accordance with published criteria*” [3]. ARVC can then be considered as a genetically determined primary cardiomyopathy, generally transmitted by the hereditary route, and attributable in about 50–60% of cases to mutations in genes which encode for cardiac desmosomes [3,10,11]. Its prevalence has been estimated at 1:1000 to 5000 and so it is considered a rare disease, developing more frequently before the age of forty and in athletes, with sudden death as the first clinical manifestation [3,6]. 

The pathology seems to more frequently affect male subjects, with a ratio estimated at 2–3:1, while there does not appear to be a significant difference between the two sexes for asymptomatic carriers. Regarding the prognosis, however, it seems to be more unfavorable for male subjects [12]. Generally, symptoms tend to occur more frequently in subjects between 30 and 45 years of age, while they tend to be less frequent in adolescents and even more so in children [13]. It is important to underline that ventricular arrhythmias could arise in an early phase of the disease, even several years before structural alterations become evident [9]. It tends to manifest clinically with palpitations, both intermittent and sustained, and/or with syncopal events, up to cardiac arrest and sudden cardiac death, before showing any other symptoms [14]. Therefore, the aim of this review is to systematically analyze the post-mortem/forensic literature and autopsy findings to describe the macroscopic, microscopic and radiological characteristics of ARVC, and then to focus on the increasingly widely used molecular analyses, such as post-mortem genetic tests, evaluating both their great diagnostic potential and their importance for prevent family members from suffering from the disease. 

## 2. Materials and Methods

An evidence-based systematic review of the literature was conducted according to the PRISMA guidelines to evaluate the state of the art of the post-mortem diagnostic techniques for correctly identifying arrhythmogenic right ventricle cardiomyopathy. The search was performed using the electronic databases PubMed and Scopus. The search was expanded by screening the reference lists of the articles that were eligible for inclusion. A pre-selection of the articles was conducted based on specific inclusion criteria: “English language” and “full text availability”. The following search queries were used: (i) Arrhythmogenic Right Ventricular Cardiomyopathy AND forensic AND genetic AND molecular autopsy AND gene mutation; (ii) arrhythmogenic right ventricular cardiomyopathy AND sudden cardiac death AND forensic; (iii) Arrhythmogenic Right Ventricular Cardiomyopathy AND forensic AND molecular autopsy; (iv) Arrhythmogenic Right Ventricular Cardiomyopathy AND post-mortem genetic test. The last date searches were performed was 20 November 2023. According to the PRISMA guidelines, the identified records were independently evaluated by three of the authors. After the removal of duplicates, the titles and abstracts were screened and those considered not relevant to the review were excluded. 

The data extraction was performed by two investigators and then verified by two other authors.

## 3. Results

**Figure 1:**
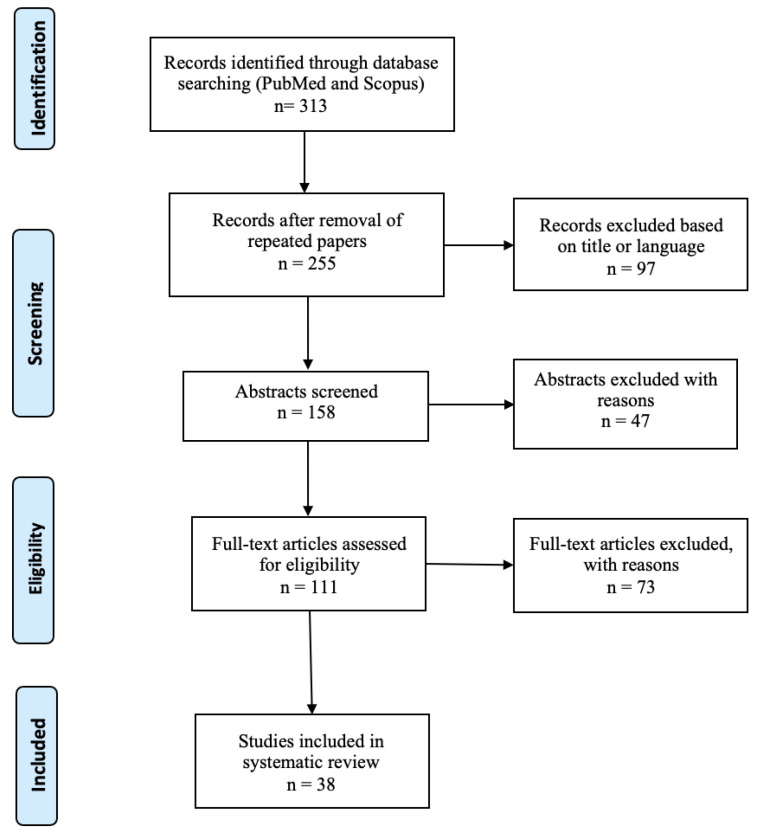
Flowchart of literature selection process.

Figure 1 shows the results of the paper selection. The selection process led to the inclusion of 38 articles including case reports, case series and reviews. 

### 3.1. Risk of Bias

The included studies were published over a long time period; thus, despite our efforts to uniformly evaluate the existing literature, the data obtained from the study should be interpreted while considering the recently acquired knowledge. It is also important to highlight that the genetic data could have an intrinsic risk of bias due to the different gene analysis panels (i.e., number of genes that are screened) that were used by each research group. 

### 3.2. Gross Examination

The main findings of ARVC are adipose or fibro-adipose replacement of myocytes associated with ventricular atrophy [8,10,14,15,16,17,18,19,20]. These patterns are predominantly described in the right ventricle, where the most common affected regions are referred to the so-called “triangle of dysplasia”: it is formed by the right ventricular inflow tract, apex and outflow tract [14]. Despite that, the disease can only involve the left ventricle or both ventricle (biventricular forms or left ventricular predominant pattern) [10,15]. In particular, Mansueto et al. [16] reported that right-dominant ARVC represents about 39% of the cases, the dominant-left form (arrhythmogenic left ventricular cardiomyopathy: ALVC) the 5%, and the biventricular form in the 56% of the cases. Corrado et al. [15] described a left ventricle involvement in about 76% of the cases, although it usually limited to subepicardium or midmural layers of the free wall. The affected chamber shows, typically, global or regional dilatation and/or a wall thinning due to both the reduction of myocytes and the increase in adipose and fibro-adipose tissue [14,17,18]. It is reported that wall thinning due to myocardial atrophy usually progress over the time, starting from the epicardium and, then, extending toward the endocardium [15,19,20]. The disease progression can lead to aneurysmal dilatation of the ventricular cavities, severe segmental dilatation and remodeling processes with formation of fibrotic areas [10,16,17,18,19,20].

### 3.3. Histological Findings

**Figure 2:**
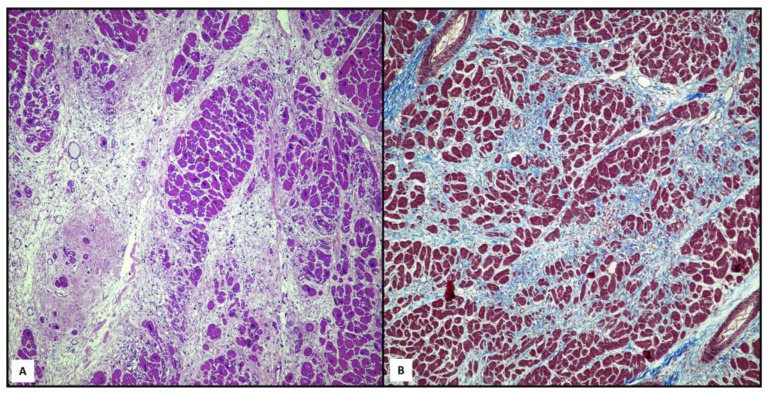
Cardiac tissue from 16-year-old boy, who suddenly died after a soccer game. Histopathological features reveal an extensive myocardial atrophy, myocytolysis and fibro-fatty replacement ((**A**), hematoxylin–eosin, magnification: 20×). Masson trichrome stain confirmed the significant fibro-fatty component (blue color) surrounding the residual cardiomyocytes ((**B**), magnification: 20×). (Contributed by A. Ieni, Messina, Italy.)

Although the consequences of adipose and fibro-adipose replacement can be detectable during gross examination, the literature has described several cases of cardiac deaths, later classified as ARVC, in which there was no macroscopic evidence of the pathology and was subsequently identified during the histological examination [4,7,9,11]. The most commonly encountered histological findings are represented by fibro-adipose replacement of myocytes, typically localized at the triangle of dysplasia: free wall of the right ventricle, especially posteriorly, followed by an involvement of the outflow tract and the apex (Figure 2A,B) [16,17,18]. A residual cardiomyocyte number < 60% evaluated by a morphometric analysis, with fibrous replacement of the right ventricular free wall myocardium are reported as the main diagnostic criteria [14]. The myocardiocyte replacement usually originates at the subepicardial level, and then tends to extend to the rest of the wall until it reaches the endocardium [21,22,23,24,25]. In the literature, cardiac wall fatty infiltration alone is not considered as a sufficient morphological hallmark of ARVC [10,15,20]. However, some authors highlighted that the mere replacement of myocytes with adipose tissue, in the absence of fibrotic phenomena, would seem to be associated with the thickening of the ventricular walls, the complete sparing of the left ventricular wall and the little or complete absence of an inflammatory infiltrate [21,22,23]. The left ventricle involvement is increasingly frequent, and it is usually associated with the sparing of the septal wall [11]. Myocyte degeneration is observed microscopically, which is related to signs of adipogenesis, which are all consistent with the model of myocyte damage and healing. Several researchers described the presence of inflammatory infiltrates as a common feature [7]. In particular, multifocal interstitial concentrations of mononuclear cells (mainly T lymphocytes) located near necrotic or damaged cardiomyocytes have been found [14,15,16,17,18]. These inflammatory infiltrates can be observed in both ventricular free walls, even in hearts with macroscopically right-ventricle-dominant disease [14]. There is also an association between the fibro-adipose infiltrate and a greater involvement of the left ventricular wall and between inflammatory infiltrate and myocardial atrophy [16,17]. Mansueto et al. [16] also described perivascular fibrosis and areas of hypertrophy and/or areas of coagulative necrosis as signs of hypoxic damage in the first stage of the disease.

### 3.4. Radiological Findings

The main post-mortem radiological techniques are represented by post-mortem computed tomography (PMCT), Post-Mortem Computed Tomography Angiography (PMCTA) and post-mortem magnetic resonance (PMMR), which are considered particularly useful in the diagnosis of cardiac pathologies, especially in case of SCD [26]. Puranik et al. [26] identified five cases of ARVC using PMMR, but the diagnosis was not confirmed in one case at autopsy (one false positive). In the diagnosis of cardiac morpho-structural pathologies, MRI has both a higher sensitivity and positive and negative predictive values than CT. Detecting alterations in the myocardial wall with T2-weighted images; the evaluation of the right ventricle (RV), left ventricle (LV) and right ventricle outflow tract (RVOT) area; and the subsequent comparison with reference models could be particularly useful for reaching a correct diagnosis [27,28]. Furthermore, an increase in the ratio between the area of the right ventricle to the left ventricle was documented in subjects with an autopsy diagnosis of ARVC (RV/LV = 2.2 ± 0.3) compared to unaffected subjects (RV/LV = 1, 1 ± 0.4, *p* = 0.0002) [26]. Post-mortem studies comparing histological findings and cardiac post-mortem magnetic resonance (CPMMR) with late gadolinium enhancement (LGE) have highlighted a correspondence between the areas of fibro-adipose replacement and the areas with gadolinium enhancement [28].

### 3.5. Genetics

The main genetic findings have been summarized in Table 1.

In the literature, post-mortem genetic tests are being increasingly used to reach a correct ARVC diagnosis [29,30,31,32,33,34,35,36,37,38]. The examined literature confirmed that the genes with strong evidence are represented by PKP2, DSG2, DSC2 and DSP [29,30,31,32,33,35,36,37,38]. The most frequently observed mutations are in the PKP2 gene [31,32,35,36,37]. Pathogenic or likely pathogenic variants have been described in DSG2 [30,36], PKP2 [31,36,37] and DSP [37]. Furthermore, new variants described as pathogenic have been reported in the PKP2 gene (187_188insC; 1925–1927 ACA del) and DSC2 (p.Gly790Arg) gene [31,33]. Studies have highlighted SCD cases with a negative ARVC phenotype, in which pathogenic mutations related to arrhythmogenic cardiomyopathy were identified: PKP2 648_651delATAC, PKP2 1618 G>A, PKP2 1843T>A and DSG2 p.P927L [31,36]. Cases of morphological diagnoses of ARVC have also been described with benign variants: DSP R2639Q, DSP V1639M, DSP A206T, PKP2 76G>A and PKP2 805G>A [29,31].

Mutations in the PKP2 gene have also been associated with the onset of other pathologies, such as Brugada syndrome (BrS) [32]. Furthermore, mutations in genes commonly associated with other cardiomyopathies, such as MYBPC3 Glu1179Lys and LMNA p. T621M, were detected in a case with cardiac structural alterations typical of ARVC [34].

Similar mutations in genes related to the onset of other pathologies, such as SCN5A p.V1597M, have been identified in subjects with an ARVC cardiac phenotype [38].

### 3.6. Genetic Counselling and Family Screening

First-line genetic tests should be aimed at identifying genes associated with the presented phenotype [39]. In the case of negative results, more extensive sequencing or analyses may be indicated [40]. If a pathogenic/likely pathogenic (P/LP) variant is identified in a living person, their first-degree relatives should be screened for that causal variant [41]. If the mutation is identified in a deceased person, screening should also be extended to their close relatives. Genetic tests are not indicated if a variant of uncertain significance (VUS) is found [3,39,40,41,42,43,44].

In the case of polygenic cardiomyopathies, new screening methods are still being validated; among these, polygenic risk scores (PRS) could gain importance in the near future [3].

## 4. Discussion

Arrhythmogenic right ventricular cardiomyopathy is a genetic disorder that can develop in individuals of any age, although it is more frequently observed between the second and fourth decades of life, representing a possible cause of SCD in young people. Due to the great phenotypic and clinical variability of the pathology, a post-mortem diagnosis based on macroscopic and microscopic findings is not always possible [14].

Therefore, this review aims to describe in detail the pathway for ARVC diagnoses, analyzing the macroscopic and microscopic autopsy findings, as well as the fundamental contribution of post-mortem instrumental investigation. Furthermore, the review highlights the importance of performing post-mortem genetic tests, as the identification of known pathogenic gene mutations could not only allow us to reach the correct diagnosis, especially in patients with a negative phenotype, but it would also be useful for prompting screening programs for the family members of the deceased.

### 4.1. Macroscopical Findings

ARVC is morphologically characterized by the progressive replacement of myocardial tissue with adipose or fibro-adipose tissue, which is frequently evident histologically and, sometimes, also macroscopically [13]. As mentioned before, historically, it was believed that this pathology almost exclusively affected the right ventricle, although today, it is known that its phenotypic expressions are quite heterogeneous, as it can involve the right ventricle only (39% of cases), the left ventricle (5%), or both (56%) [16]. The replacement could start from both the epicardium and myocardium up to the full thickness of the wall [22,23]. Therefore, during gross examination, it is possible to highlight clear alterations in the cardiac walls, as the presence of whitish or yellowish areas in the myocardial wall up to the formation of single or multiple aneurysms, mainly located in the so-called “dysplasia triangle” (consisting of the outflow tract, apex and infero-basal or subtricuspid region of the right ventricle) [14,22]. Instead, the left ventricle is usually involved at subepicardial or medial layer of the free wall [15].

### 4.2. Microscopical Findings

Several cases where the adipose and fibro-fatty replacement of cardiomyocytes is not visible on gross examination have been described; despite that, in some of these cases, the pathology is still identifiable through histological examination [12]. The most common histological findings are represented by adipose, fibrous or fibro-adipose infiltrations of the cardiac walls; among these, the most common is represented by the fibro-adipose replacement of myocytes, frequently detectable at the so-called “dysplasia triangle” [17]. Furthermore, the residual myocardiocytes located in the areas surrounding the fibro-adipose degeneration may appear partially hypertrophic or atrophic, or even vacuolated, with the possibility of detecting coagulative necrosis and inflammatory infiltrates, mostly formed by lymphocytes [16]. Some authors have then attempted to quantify the percentage of fibrous and/or fibro-adipose tissue replacement necessary to diagnose the pathology, identifying a ventricular wall infiltration of at least 3% of adipose tissue and over 40% of fibrous tissue, by evaluating seven microscopic fields at a 400× magnification and with the aid of a diagram analysis system [16,45]. Furthermore, it was reported that a myocardial wall adipose infiltration percentage between 5 and 20% should lead to a suspicion of ARVC [46]. Hagemeier et al. [47] have proposed a digital microphotograph method to better determine the percentage of myocardial wall fat and connective infiltration: pathologists should identify the myocardial wall fibro-fatty infiltrations in at least seven fields of view in five different myocardial regions, using Elastica van Gieson and Sudan III staining.

More specifically, this percentage range was introduced to facilitate ARVC diagnosis through the identification of adipose and connective tissue transmural replacement, which is frequently associated with nests of adipose cells that are detectable in localized areas of the right ventricular wall [17]. Recently, the Heart Rhythm Society (HRS) has identified specific major and minor criteria for ARVC diagnoses, including clinical characteristics, family history, imaging, and histological analysis of specimens, aimed at determining the right ventricular fibrotic replacement rate [17]. Regarding histopathological characteristics, the major diagnostic criteria involve the finding of <60% residual myocytes through a morphometric analysis and/or the fibrous replacement of the right ventricle myocardium free wall, with or without adipose tissue replacement, in at least one endomyocardial biopsy specimen [18]. Instead, the minor criteria include the finding of 60 to 75% residual myocytes through a morphometric analysis and/or fibrous replacement of the right ventricle myocardium free wall, with or without replacement of adipose tissue, in at least one endomyocardial biopsy specimen [18].

The possibility of using immunohistochemistry (IHC) and Western blotting (WB) has also been documented for evaluating the reduction in proteins in cardiac tissue, usually due to the presence of gene mutations that do not allow for their correct formation, which underlies the pathology itself [24,25]. Hung et al. described cases of ARVC diagnosed using these methods; they found a decrease in the expression of αT-catenin and plakophilin-2 (PKP-2) as a consequence of a CTNNA3 gene mutation, although it is not commonly involved in the pathology onset [24]. αT-catenin is a cytoplasmic protein essential for cytoskeletal remodeling; it interacts with the desmosomal protein plakophilin-2 in the cadherin–catenin complex [24,48,49,50]. The study showed how, after knocking down the CTNNA3 gene, which encodes αT-catenin, the expression of plakophilin-2 was reduced too [48]. Therefore, since PKP2 is one of the most important protein in ARVC pathogenesis, it has been possible to demonstrate how mutations in genes that encode for specific proteins are capable of indirectly altering other proteins, producing the same results [50]. In particular, the alteration of αT-catenin, as well as PKP2 and Connexin 43, is associated with electrical conduction abnormalities, ventricular arrhythmias and sudden cardiac death [49]. Moreover, further studies conducted with Western blotting have produced results similar to those obtained with IHC [49]. Nevertheless, the use of these methods for the evaluation of reductions in protein levels is not considered sufficient to reach a certain diagnosis, either in living people after performing an endomyocardial biopsy or in a corpse after performing autopsy investigations. Therefore, in the literature, these techniques are proposed as integrative tests to reach an ARVC diagnosis [50,51,52].

### 4.3. Radiological Imaging

Radiological imaging also plays an important role in ARVC identification, especially using cardiac MR [53]. The literature describes cases of fibro-adipose infiltration and replacement of myocardial tissue that were identified using high-spatial-resolution spin-echo MR images and late gadolinium enhancement (LGE). It has been demonstrated that LGE of ventricles corresponds to the replacement of fibrous adipose tissue, which is frequently detected in the subepicardial region [28,54].

There are great differences between the cardiac imaging performed in living people and post-mortem; in the first case, it is possible to acquire information regarding both the morphology and the kinetics of the cardiac chambers, mainly through exploiting the use of contrast, whereas, post-mortem imaging does not provide functional, but only morphological information [26]. Furthermore, although in absence of movement artefacts, it is possible to find traits attributable to post-mortem transformative phenomena, such as putrefaction or due to the variation in body temperature [26]. The post-mortem radiological diagnosis of cardiomyopathies makes use of PMCT, PMCTA and PMMRI, although their application is still highly debated [27,55,56,57,58,59,60,61,62,63].

In cases of sudden cardiac death occurring in young subjects, MR has been proven to be particularly useful when it has not been possible to perform a traditional autopsy. Furthermore, especially in cases of SCD in adults, it has shown higher levels of diagnostic accuracy compared to CT examination [26].

Roberts et al. [60] conducted a study where it was shown that CT was unable to accurately define any of the intracardiac pathologies evident on MR imaging, such as ARVC. Despite this, CT remains the most used and the most easily accessible technique in the forensic field [57]. The use of PMCTA has also been considered in the evaluation of ARVC, albeit with poor results. It is known that this method exploits two different methods of administration of the contrast: it can be inoculated into the aorta before the autopsy or directly into the coronary arteries after the removal of the heart at autopsy [56,59]. This test has proven to be useful in the evaluation of coronary artery disease, with better results than the CT exam, but has also been proven to be less useful in characterizing structural pathologies affecting the heart walls [27,60].

Taylor et al. [53] conducted an analysis on a series of 400 post-mortem cases in children using T1/T2-weighted MR imaging (STIR), finding a high sensitivity, specificity and both positive and negative predictive values in the identification of structural pathologies of the cardiac wall. Among these alterations, fatty infiltrations of the ventricular walls and thinning of the apical wall are the most commonly described [28,55,62].

To better diagnose ARVC, Marcus et al. [57] evaluated the right and left ventricle area and of the right ventricular outflow tract of 108 individuals. In the absence of other pathological conditions capable of determining its onset, an area of RV greater than at least twice the LV area, evaluated on four-chamber MR images, or a regional dilatation in RVOT could be used to diagnose ARVC. Three false positive cases and no false negatives were described.

Jackowski et al. [27] proposed analyzing the dimensions and weight of the heart right and left ventricular masses on PMMR to evaluate hypertrophy or dilation phenomena, and subsequently to be able to perform a comparison with reference values of normal hearts.

A comparative study has also been proposed to observe the main differences between a normal heart weight quantified with PMMR and the values subsequently obtained during the autopsy examination. The PMMR evaluation was considered more precise, as during the autopsy, the heart is removed and weighed with valve and vascular structures, which can instead be excluded if the same evaluation is performed during a radiological examination [56]. Ultimately, among the radiological methods performed post-mortem, PMMR is nowadays considered the one of greatest value. Conversely, the PMCT and PMCTA methods are considered of less use in characterizing this pathology.

### 4.4. Post-Mortem Genetic Tests

Macroscopic, microscopic and radiological abnormalities can lead to a correct diagnosis of ARVC, but unfortunately, they are not always detectable. In fact, phenotype-negative ARVC forms have been described [19]. In this particular category, in the absence of even histological morphostructural alterations, the pathology first manifests with SCD, frequently secondary to the development of ventricular arrhythmias; to correctly identify these forms, a genetic analysis should be performed, leading to the identification of specific gene mutations [19,56].

As mentioned, ARVC is a hereditary genetic pathology transmitted in more than 50% of cases through an autosomal dominant route and with variable penetrance, and it is influenced by age, gender and physical activity [3]. Moreover, the literature describes forms transmitted by an autosomal recessive route, which are often associated with syndromic conditions and polygenic forms [29,55]. Sporadic forms have also been described [55]. Historically, Naxos disease, a recessive form of ARVC characterized by heart involvement associated with palmoplantar keratosis and woolly hair, made it possible to focus on the structures, and therefore on the genes, involved in the onset of this pathology [64,65].

At the beginning, genes that encode for cytoskeletal or sarcomeric proteins (involved in the onset of HCM) were studied, with poor results. Naxos disease allowed us to observe the structures shared by epidermal cells and myocytes—desmosomes and zonulae adherens—and subsequently identify genes that are potentially responsible for the onset of the pathology [64]. As reported in The Guidelines for the Management of Cardiomyopathies of the European Society of Cardiology (ESC) of 2023, pathogenic (P) or likely pathogenic (LP) gene variants can be identified in up to 60% of patients with a diagnosis of ARVC [3]. These genes can be distinguished into two different classes: (i) genes that encode for desmosomal proteins and (ii) genes that encode for non-desmosomal proteins. The first class is responsible for the onset of more than 70% of the total forms of ARVC described in the general population, while the second one represents the remaining percentage, which is certainly less common [30,31,32,33].

**Figure 3:**
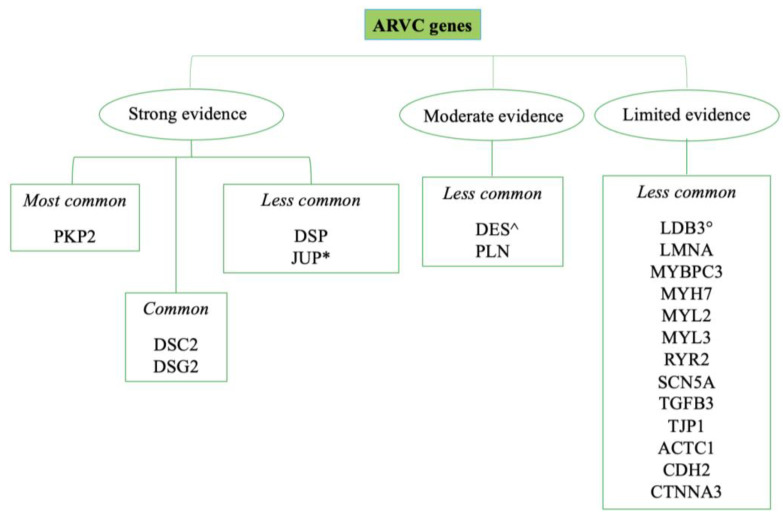
Frequency and strength of evidence of genes’ association with ARVC [3]; most common: >10% of the cases; common: 1–10% of the cases; less common: <1% of the cases; *: genes associated with Naxos disease; ^: desminopathy; °: myofibrillar myopathy.

Among the genes encoding for desmosomal proteins, the most involved are desmoplakin (DSP), plakophilin-2 (PKP2), desmoglein-2 (DSG2), desmocollin-2 (DSC2) and plakoglobin (JUP). The non-desmosomal proteins include transmembrane protein 43 (TMEM43), ryanodine receptor 2 (RYR2), desmin, lamins A and C, striatin, titin (TTN), transforming growth factor-b3 (TGFb3) and catenin alpha-3 (CTNNA3) [30,32,34,35,36] (Figure 3). For each of the mutations found, the ESC has recently proposed a classification to distinguish them in relation to the strength of their association with the onset of the pathology [3].

Therefore, given the great importance of genetic evaluations in reaching a correctly ARVC diagnosis, it is essential to evaluate the pathogenicity of each variant found [66]. According to the American College of Medical Genetics and Genomics and the Association for Molecular Pathology guidelines, each mutation found after performing genetic tests should be distinguished as pathogenic, likely pathogenic, of uncertain significance (VUS), probably benign or benign [67]. Because of the continuous increase in the number of variants described in the literature, performing a post-mortem genetic analysis is of fundamental importance to correctly identify the cause of death in all the cases of ARVC without any evident cardiac structural alterations [68,69,70].

PKP2 gene mutations, which are responsible for the onset of the pathology in 11–43/51% of cases, are the most frequently found, followed by mutations in the DSG2 (3–20%), DSP (1–7%) and JUP (0.5–16%) genes [31,32,71]. Mutations in genes that encode for non-desmosomal proteins are less represented [71].

There is limited evidence for genotype–phenotype associations, probably due to the high variability of the mutations themselves [30]. At the same time, several cases of mutations in the main genes responsible for the onset of the pathology and negative phenotype forms have been described, where death occurred mainly due to malignant arrhythmias, particularly ventricular fibrillation [31]. It has also been described that the ventricular disfunction occurring in arrhythmogenic cardiomyopathy can vary in relation to the genetic variants. The Heart Rhythm Association [17] reported a dominant pattern of right-dominant cardiomyopathy associated with genes encoding desmosomal, intercalated disk and ion channel proteins (e.g., PKP2, JUP and DSC2), and a left-dominant involvement associated with mutations in genes encoding cytoskeletal, sarcomeric, ion channel and mitochondrial proteins (e.g., LMNA, DSP, LBD3, desmin). In particular, some researchers showed an association between DSP mutations and the biventricular or predominantly left ventricular pattern [14,72,73].

Sato et al. [29] described three cases with a PKP2 gene mutation and negative phenotype, in which the diagnosis of the pathology was reached after performing genetic analyses. Among these, the G-to-A mutation of DSP codon 2639 c.7916G>A has been associated with no cardiac muscle alterations at both the macroscopic and microscopic levels. Despite this, Yu et al. [70] described the same mutation in a subject with a severe dilation and dysfunction of the right ventricle. This finding allows us to see that the typical alterations in ARVC are not always unequivocally attributable to the same mutation.

Similarly, Zhang et al. [31] described six SCD cases with a post-mortem identification of mutations in the PKP2 gene, with no cardiac alterations. This has led to the belief that there may not be a direct causal correlation between alterations in desmosomal proteins and ARVC pathophysiology, but that alterations in desmosomal proteins are themselves capable of predisposing to arrhythmias even before fibro-adipose replacement occurs. Larsen et al. [74] performed genetic analyses on 14 subjects with morphostructural cardiac alterations attributable to ARVC, and found a mutation in the PKP2 gene in only one case.

Furthermore, in the literature, associations between ARVC gene mutations and the onset of other pathologies have been described [32]. Similarly, cases of a clear ARVC phenotype and mutations in genes typically associated with other cardiomyopathies have been described [34]. In the cardiac muscle, desmosomes are components of the intercalated discs and play a key role in the correct functioning of the myocardial syncytium. However, desmosomal proteins also interact with other structures, such as ion channels, and allow for electrical impulse genesis and transmission. Among the desmosomal proteins, plakophilin-2 is known to interact with the voltage-gated sodium channel complex. Therefore, PKP2 gene mutations can alter the functioning of sodium channels, and so the onset of arrhythmias [75]. This protein interacts with Nav1.5, which is normally encoded by the SCN5A gene. Other studies conducted in rats confirmed a sodium current (INa) reduction after PKP2 knockdown [76,77].

Huang et al. [32] described a PKP2 gene mutation that led to a functional alteration in sodium channels, which is responsible for the onset of Brugada syndrome (BrS). Cerrone et al. [78] conducted genetic tests on 200 subjects affected by BrS with no mutations in known genes usually related to this pathology nor signs of ARVC. PKP2 gene mutations were detected in five of these subjects, further demonstrating the difficulty in identifying a genotype–phenotype correlation.

Choung et al. [34] described a heterozygous mutation for a single nucleotide (SNP)—a missense variant of unknown significance (G->A, Glu1179Lys)—in the MYBPC3 gene, in a subject with fibro-adipose degeneration mainly affecting the right ventricle, although this gene mutation is predominantly responsible for the onset of HCM.

The high variability of gene mutations and their heterogeneous phenotypic manifestations highlight the difficulty of correctly defining the pathophysiological mechanisms underlying the pathology [35,78]. At the same time, it is evident the importance of performing a molecular autopsy, especially in those cases of SCD with no macroscopic and microscopic alterations; this could lead to make a diagnosis that would be otherwise difficult to reach and allow us to implement genetic databases for more targeted post-mortem genetic analyses [37,38,79].

Therefore, although some gene mutations are associated with more common phenotypic manifestations have been described, there are many cases where it is not possible to identify a genotype–phenotype correlation because of the high variability of mutations affecting different codons or single nucleotides in the same gene [19,33,37,38,80].

### 4.5. Genetic Counselling and Family Screening

The post-mortem assessment of gene mutations is of fundamental importance for starting family screening and genetic counseling programs.

Family screening and genetic counseling have a dual purpose: (i) the early identification and treatment of the disease, and avoiding or delaying the occurrence of all related complications, such as sudden cardiac death, and (ii) to provide not only medical but also psychological support to all those subjects with a positive test for pathogenic (P) or likely pathogenic (LP) variants, and, eventually, to their family members [3,39,40,41,81].

Most of these tests focus on monogenic variants, despite knowing that some polygenic forms can occur, and they are not always identifiable [3,42]. Moreover, according to the characteristics of the mutated genes, the pathology could develop with variable penetrance, leading to important differences in phenotypic expressions, depending on the age of onset [42,82].

Moreover, the post-mortem identification of P/LP mutations requires the application of screening programs which are different from those envisaged for relatives of subjects [40,41,43,83]. It is known that mutations identified in individuals who are still alive requires screening of their first-degree family members, whereas if the same mutations are identified in deceased subjects, screening is extended to second-degree relatives [3].

If the common screening tests do not allow for the identification of known mutations, it is possible to assume that (i) it is not a known monogenic variant, (ii) the pathology is not genetically based, and (iii) it is a polygenic form where the effects of multiple gene mutations contribute to the onset of the pathology. In the latter case, the risk of genetic transmissibility is reduced and, therefore, if at an initial clinical evaluation of relatives, there are no particular clinical signs suggestive of the pathology, continuous surveillance could also be avoided [42,43,44,84]. Recently, it has been proposed that PRS could be used for a simultaneous evaluation of different genomic variants that individually contribute to the development of the pathology and the calculated score would constitutes the aggregate risk [3,43,85]. If no P/LP variants are identified, genetic testing of relatives is not recommended, only clinical follow-up [3]. Hence, the importance of performing post-mortem genetic tests for both (i) carrying out studies aimed at identifying such mutations, which, if correctly highlighted, could allow researchers to directly screen the relatives of deceased subjects, and (ii) to identify mutations that have not yet been described, or reconsider those of unknown significance (VUS), to contribute to the implementation of international databases [3,44,83].

## 5. Conclusions

**Figure 4:**
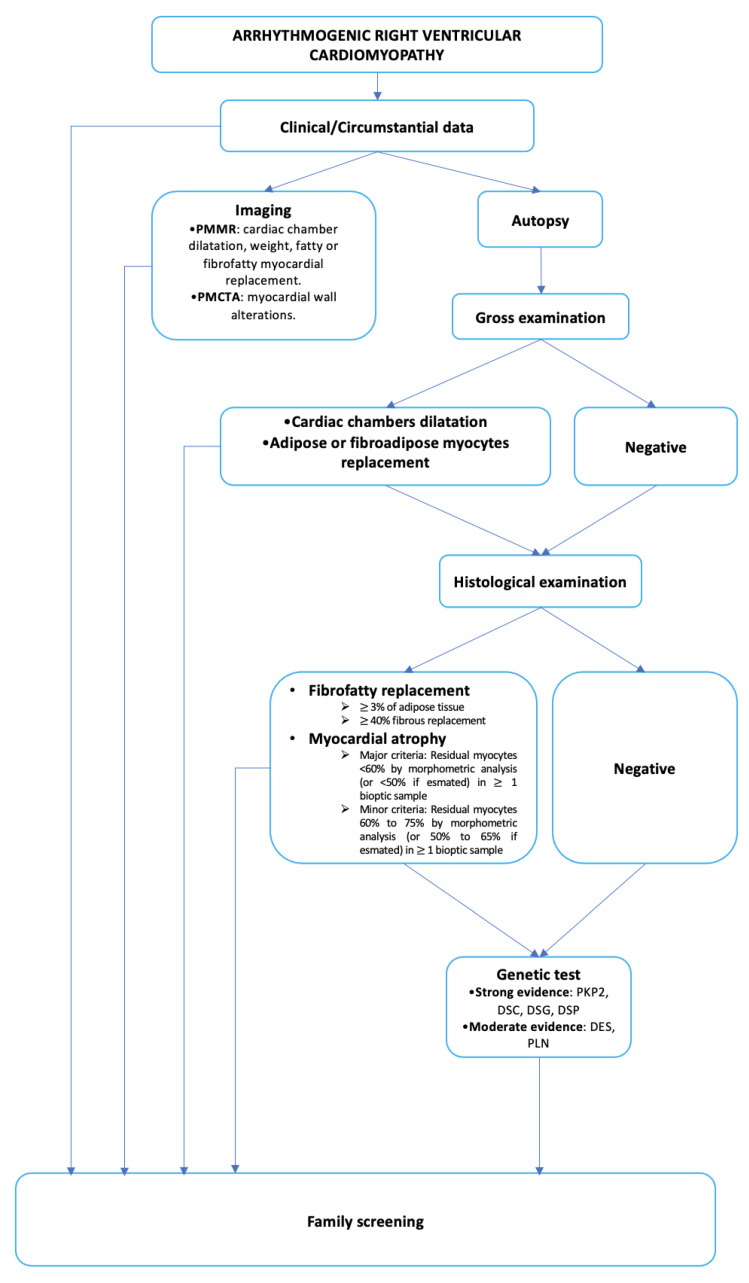
Synthesis of the main investigations both to reach a correct post-mortem diagnosis of ARVC and to guarantee family screening.

In conclusion, the activity of clinical and forensic pathologists in ARVC diagnoses is of pivotal importance, not only to correctly identify the cause of death, but also to make it possible to carry out screening programs on the relatives of the deceased. It has been highlighted that it is not possible to reach a correct diagnosis during autoptic investigation, through macroscopic and microscopic examination, because of the presence of negative phenotype forms; in all these cases, a genetic analysis could orient towards, or even be decisive in, reaching the correct diagnosis. Furthermore, as mentioned before, the possibility of an ARVC post-mortem diagnosis in those subjects with a negative medical history could lead to the start of screening programs on their relatives to avoid other cases of sudden cardiac death (Figure 4).

The differences in the application of post-mortem molecular tests in clinical or judicial autopsies must also be highlighted. During judicial forensic investigations, it is not always possible to perform post-mortem genetic tests both due to the high costs and because it is not always considered necessary and useful by the judicial authority. For this reason, further studies should be conducted to better define the genes that are more frequently responsible for the onset of this pathology to provide a specific panel to make this genetic analysis cheaper.

## Figures and Tables

**Table 1:** Genetic results. SCD: sudden cardiac death. SUNDS: sudden unexpected nocturnal death syndrome. m: male. f: female. NR: not reported. P: pathogenic variant. LP: likely pathogenic variant. hpP: highly possible pathogenic variant. VUS: variant of unknown significance. D: damaging variant. B: benign.

Authors	Year	Sample (n)	Gross/Microscopic Data	Type of Sample	Gene/Nucleotide or Amino Acid Change	Type of Mutation
Sato T. et al. [29]	2015	15 SCD (23.4 ± 8.8 years)	-/-	Blood	Case 1: DSP/R2639Q (c.7916G>A)	B
			-/-		Case 2: DSP/V1639M (c.4915G>A)	Novel—B
			-/-		Case 3: DSP/A206T (c.616G>A)	Novel—B
Cittadini F. et al. [30]	2014	1 (41 year)	+/+	Blood	DSG2/p.Val920Gly (c.2759T>G)	P
Zhang M. et al. [31]	2016	25 ARVC and 25 SCD	+/+ and -/-	Heart	Case 1 (ARVC): PKP2 187_188insC	Novel—D
					Case 2 (ARVC): PKP2/76G>A	B
					Case 3 (ARVC): PKP2/1951C>T; 76G>A	D; B
					Case 4 (ARVC): PKP2/2062T>C	D
					Case 5 (ARVC): PKP2/805G>A	Novel—B
					Case 6 (ARVC): PKP2/1925–1927 ACA del	Novel—D
					Case 1 (SCD): PKP2/1993C>T	Novel—B
					Case 2 (SCD): PKP2/1592T>G	B
					Case 3 (SCD): PKP2/1016 T>C	B
					Case 4 (SCD): PKP2/648_651delATAC	D
					Case 1 (SCD): PKP2/1618 G>A	D
					Case 1 (SCD): PKP2/1843T>A	D
Huang L. et al. [32]	2016	119 SUNDS (18 to 52 years)	-/-	Blood	Case 1: PKP2/p.Ala159Thr (c.475G>A); p.Gly265Glu (c.794G>A); p.Thr723Thr (c.2169A>G)	NR
Mak C.M. et al. [33]	2019	10 SCD	+/+	Blood	Case 1: DSC2/p.Gly790Arg (c.2368G>A)	Novel—P
					Case 2: No Molecular Autopsy findings	
					Case 3: No Molecular Autopsy findings	
Choung et al. [34]	2017	1 SCD	+/+	NR	MYBPC3 p. Glu1179Lys	VUS
Lahtinen et al. [35]	2013	112 subjects	NR	Blood	PKP2/p.Q59L (n. 85 cases)	NR
					PKP2/p.Q62K (n. 12 cases)	
					DSG2/p.3059_3062delAGAG (n. 5 cases)	
					DSP p.T1373A (n. 10 cases)	
Hata Y. [36]	2015	25 SUDS	-/+	Blood	Case 1: DSG2 p.R824C	P
			-/+		Case 2: PKP2 p.P717L	hpP
			-/+		Case 3: JUP p.A143T	hpP
			-/-		Case 4: DSG2 p.P927L	hpP
Junttila M.J. [37]	2018	96 SCD	NR	Heart	Case 1: PKP2_1114G>C	LP
					Case 2: DSP_2422C>T	LP
					Case 3: DSP_6295-6296CC>AT	VUS
					Case 4: DSP_6307A>G	VUS
					Case 5: DSG2_2906C>T	VUS
Hertz C.I. [38]	2015	72 SCD (n. 14 ARVC)	+/+	Blood	Case 1: SCN5A/p.V1597M (c.4789G>A)	NR
					Case 2: LMNA/p. T621M (c.1862C>T)	
					Case 3: LDB3/p. Q157P (c.1550A>C)	
					Case 4: DSP/p. N1306S (c.3917A>G)	
					Case 5: DSP/p. R925Q (c.2774G>A)	

## Data Availability

All the data are reported in the paper.
